# Optimizing the encapsulation of the refined extract of squash peels for functional food applications: A sustainable approach to reduce food waste

**DOI:** 10.1515/biol-2025-1124

**Published:** 2025-07-11

**Authors:** Rim Ben Mansour, Hanen Falleh, Majdi Hammami, Feriel Ben Hadid, Lilian Barros, Neji Tarchoun, Spyridon A. Petropoulos, Riadh Ksouri

**Affiliations:** Laboratoire des Plantes Aromatiques et Médicinales, Centre de Biotechnologie de Borj Cedria, BP 901 - Hammam Lif, 2050, Tunis, Tunisia; Centro de Investigação de Montanha (CIMO) Instituto Politécnico de Bragança, Campus de Santa Apolónia, 5300-253, Bragança, Portugal; Laboratório Associado para a Sustentabilidade e Tecnologia em Regiões de Montanha (SusTEC), Instituto Politécnico de Bragança, Campus de Santa Apolónia, 5300-253, Bragança, Portugal; Institut Supérieur Agronomique de Chott Mariem, BP 47, 4042 Chott Mériem, Sousse, Tunis, Tunisia; Department of Agriculture, Crop Production and Rural Environment, University of Thessaly, Fytokou Street, 38446, Volos, Greece

**Keywords:** response surface methodology, antioxidant activity, biowaste valorization, refined-extract, encapsulation, antimicrobial properties, squash peel

## Abstract

Encapsulation of the refined extract from squash fruit peels was evaluated for the design of functional foods with beneficial effects on human health. The percentage of maltodextrin and gum Arabic in the wall material as well as the concentration of the refined extract were the independent variables for the Box Behnken design, and their impact on response variables (total phenolic compound [TPC] content, DPPH activity, the size of particles, and polydispersity index [Pdi]) was evaluated. The obtained emulsions were compared based on their TPC content, as well as their antioxidant activities. Optimum conditions were as follows: maltodextrin percentage of 23.8%, gum Arabic percentage of 27.7%, and phenolic extract percentage of 48.5%. Under these conditions, the response variables were the following: TPC content of 46.01 mg of gallic acid equivalent per gram of extract (mg GAE/g of E); DPPH inhibition percentage of 64.85% at 1 mg/mL, particle size of 571.22 nm, and Pdi of 0.46. Once the emulsion was optimized, we tried to define the combination of variables that produced microcapsules with the highest TPC content and the highest antioxidant activity. Physicochemical parameters and biological activities were also assessed. Microcapsule parameters were a pH of 4.2, a viscosity of 17 mPa/s, and a turbidity of 0.412 NTU (Nephelometric Turbidity Units), and color measurements were *L* = 102.4, *a* = 3.5, and *b* = 7.8. Indeed, the TPC content was 37.2 mg GAE/g dried residue and antiradical activity against DPPH was 44.2%. The inhibitory effects of the optimized emulsion on *Enterococcus faecalis*, *Pseudomonas aeruginosa*, *Salmonella typhimurium*, and *Staphylococcus aureus* were investigated, and the results indicated the highest sensitivity (89% inhibition) for *S. typhimurium*. Our results indicate the efficiency of the optimized emulsification process for developing microcapsules of high quality as well as the potential of using them in the development of novel food and pharmaceutical products.

## Graphical abstract

**Figure:**
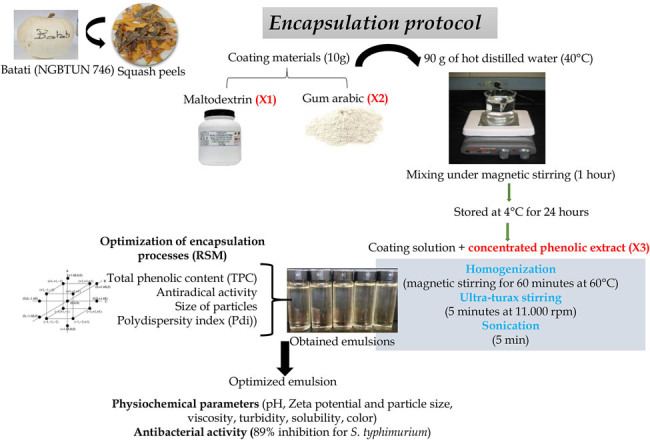

## 1 Introduction

Nowadays, fruit- and vegetable-based industries produce huge quantities of by-products which mostly remain underutilized, although they have a great economic potential [1]. These by-products present a promising chemical profile, and they could be used as raw materials for the design of integrated biorefinery approaches. Meanwhile, numerous functional compounds and platform chemicals could be developed through sequential environmentally friendly processes with various applications in the industry sector [2]. In this context, it is notable that pumpkin by-products have been commonly evaluated due to their high content in phytochemicals and macronutrients or antioxidant compounds with multiple biological properties [3,4]. It has been reported that squash peels, compared to other by-products of squash processing, contain high amounts of antioxidants, and they exhibit strong radical scavenging properties [5,6]. The enrichment of many food products with raw materials of natural origin such as phenolic compounds has received considerable interest from the food industry [7]. Phenolic compounds possess health beneficial properties, but their limited aqueous solubility hampers their bioavailability. However, stabilizing phytochemicals such as phenolic compounds is crucial due to their limitations in solubility and stability, since after their extraction from natural matrices they become susceptible to oxidation, polymerization, or condensation, especially when they are subjected to heat, light, and other oxidation factors which can reduce their value and bioactive properties [8].

By encapsulating phenolic compounds, their solubility and stability can be significantly improved, leading to enhanced absorption and bioactive potency for food and pharmaceutical products [9]. Additionally, encapsulation protects phenolic compounds from degradation, thereby preserving their structural integrity and activity [10]. Encapsulation systems also offer formulation flexibility, allowing customization for controlled release, tissue targeting, and compatibility with other ingredients. Moreover, encapsulation provides improved stability during storage and helps to maintain the targeted phenolic compound concentration and activity. Encapsulation has widely been applied to alleviate these limitations.

Comparing different combinations of encapsulating components through experimental design is important for several reasons [11]. First, it allows for the identification of the optimal formulation that provides the desired characteristics and stability for a particular application. Second, it helps understand the impact of different phase combinations on the solubility, encapsulation efficiency, and release properties of active compounds. Finally, it ensures compatibility and prevents instability.

In our previous work, we succeeded to identify the optimal extraction conditions of refined compounds from the peels of squash fruit with improved bioactivity [3]. Our results revealed that peel extracts constitute interesting antioxidant and antimicrobial agents. For that reason and in continuity with our previous study, in the present work, we aimed to optimize the encapsulation of squash-refined extract into an emulsion-based delivery system. For this purpose, different technologies of encapsulation were adopted and different ingredients were screened, focusing mainly on the ratio and composition of the coating materials. The obtained emulsions were compared based on their total phenolic compound (TPC) contents, as well as their antioxidant capacity. The optimized emulsion was further assessed for its physicochemical proprieties as well as its biological properties, mostly the antimicrobial ones. Finally, the stability of the optimal emulsion was also evaluated during a storage period of 6 weeks. With this work, we aimed to evaluate the optimal encapsulation conditions of refined squash peel extracts in order to suggest this valuable by-product as a natural matrix for the design of novel functional and healthy food products within the context of circular economy.

## 2 Methodology

### 2.1 Sample preparation and encapsulation protocol

The peels from squash landraces Batati (NGBTUN 746) were evaluated in the present study. The peel refined extract was prepared by using conventional high-energy extraction combined with ultrasound extraction, as detailed in our previous work [3]. In brief, 3 g of powdered sample were extracted using 70 mL of hydroethanolic solution (ethanol/water, 80:20) at ambient temperature under stirring for over 60 min, while the ultrasound-assisted extraction was performed using an ultrasonic bath (model: Power Sonic 405, Hwashin Technology Co., Yeongcheon-si, Republic of Korea), as described by Ben Mansour et al. [3]. Then, the obtained extracts were centrifuged at 9,000 × *g* for 15 min at 4°C, and the supernatants were subjected to vacuum-evaporation (Hei-VAP Advantage, Heidolph Instruments GmbH, Schwabach, Germany) at 45°C for ethanol removal, while the residual water was lyophilized (Christ Martin™ Lyophilisateur Alpha 1–4 LDplus NU, Germany) to dryness for subsequent analysis [3]. Maltodextrin (*X*1) and gum Arabic (*X*2) were used as coating materials (ratio 50:50) ranging from 0 to 0.8 [12]. Ten grams of coating material were dissolved in 90 g of hot distilled water (40°C) to prepare a 10% coating material solution. The solution was mixed using magnetic stirring for 1 h and then stored at 4°C for 24 h until they complete hydration. Once the coating solutions were ready, they were mixed with the concentrated extract (mg) (*X*3) and homogenized using magnetic stirring for 60 min at 60°C. The next step was the ultra-homogenization using a rotor-stator homogenizer (IKA T 25 digital ULTRA-TURRAX, Staufen, Germany) for 5 min at 11,000 rpm. Finally, the samples were sonicated for 5 min using the same equipment mentioned above.

### 2.2 Experimental design and statistical analysis

To evaluate the impact of different rates of maltodextrin and gum Arabic on phenolic compounds and their antioxidant activities, a comprehensive factorial design augmented simplex lattice, second order, was employed, with five replicates at the center point, resulting in 13 preparations [3]. The experimental layout was a three-component axial screen matrix, and the maximum coded level for all three components (*X*1, *X*2, and *X*3) was 1. The measured four responses (TPC content *Y*1 expressed as mg GAE/g of dried residue [DR]; antiradical activity *Y*2 expressed as inhibition percentage [IP]; average droplet diameters or particle size *Y*3 (*d*_3.2_ expressed in nm); and polydispersity index [Pdi] *Y*4) were inserted into the software NemrodW (LPRAI 2000, Mathieu, Nony and Phan-Tan-Luu, version 2000, Marseille-France). The experimental data were then used to reveal the relationship between each factor and the response variables with the selected regression model, using t-statistic (95% confidence interval). The terms that were non-significant (at *p*-value >0.05) were not included in the initial equation, and the data were then fitted again to the refined model. The quality of the mathematical models was assessed using the response surface methodology (RSM) and analysis of variance (ANOVA) based on the criteria previously described [3]. To maximize the content of TPCs and the antioxidant capacity, simultaneous optimization of the desirability function was also conducted.

### 2.3 Analysis of the encapsulated peel extracts

#### 2.3.1 Evaluation of the content of TPC and biological activities

***TPC*** content in the obtained extracts was assessed with the method previously described by Ben Mansour et al. [3] with slight modifications. Briefly, 100 µL of Folin-Ciocalteu’s reagent was combined with 20 µL of each emulsion and left for 5 min incubation. Then, 80 µL of CO_3_NO_2_ (75 g/L) solution were added and left for 60 min for incubation. After that, the absorbance of each sample was measured at 765 nm (EZ Read 2000, Biochrom, Cambridge, UK). The TPC was expressed in milligrams of gallic acid equivalent per gram of extract (mg GAE/g E). Analysis was repeated for at least three times.

***DPPH radical-scavenging activity*** was measured as previously described by Hatano et al. [13], using 50 µL (1 mg/mL) of extract and 150 µL of DPPH solution (200 µM). The IP% was assessed using the following formula [14]:

(1) \[\text{IP}( \% )={[}(A0\mbox{--}A1)/A0]\times 100,]\]

where *A*0 and *A*1 are the absorbance readings (at 514 nm) for the control and the sample, respectively, using a microplate spectrophotometer (EZ Read 2000, Biochrom, Cambridge, UK).

***ABTS radical-scavenging activity*** of the optimized formulation was assessed according to Re et al. [15] using 5 mL of 14 mM ABTS solution and adding 5 mL of 4.9 mM potassium persulfate (K_2_S_2_O_8_) solution. After that, the solution was further diluted with ethanol. The reaction mixture had a final volume of 1 mL, and it comprised 950 µL of ABTS^+^ solution and 50 µL of the extract. ABTS scavenging capacity was expressed as IP at 1 mg/mL.

***Antimicrobial activity*** was assessed against several pathogenic strains, namely *Staphylococcus aureus* (ATCC 25923), *Enterococcus faecalis* (ATCC 29212), *Salmonella typhimurium* (ATCC 14028), and *Pseudomonas aeruginosa* (ATCC 27853), using the protocol previously described by Falleh et al. [16]. The antimicrobial activity was expressed as growth IP.

#### 2.3.2 Emulsion physicochemical properties

***Zeta potential and particle size*** of droplets of different formulations were evaluated using a particle size analyzer (Zetasizer Nano ZS90, Malvern Instruments Ltd., Malvern, UK) [11]. For measurement of the droplet size, three repetitions were carried out.

***pH, viscosity, turbidity, and solubility*** of the formulations were assessed based on the method previously described by Falleh et al. [17].

***Water solubility index*** (WS_M_, %) of microcapsules was determined according to the modified method of Aliakbarian et al. [18] using the following formula:

(2) \[{\text{WS}}_{\text{M}}=({\text{DW}}_{\text{S}}-{\text{DW}}_{\text{M}})\times 100,]\]

where DW_S_ (g) is the dry mass of the supernatant and DW_M_ is the total dry mass of the weighed microcapsules (0.2 g).

***Color*** of phenolic microcapsules was assessed according to the method of Tao et al. [19] using a colorimeter (PCE-XXM 30, PCE Instruments UK Ltd., United Kingdom) according to the CIELAB color scale.

### 2.4 Statistical analysis

All the abovementioned tests were performed with at least three replicates. The obtained data were analyzed with the Statistical package SAS 9.1, 2002 (SAS Institute, Cary, NC, USA). The mean values of the mathematical models were compared according to the Newman–Keuls method (SNK; *p* < 0.5), while the mean values from the analysis of the encapsulated extracts were compared using the Duncan multiple range test at *p* < 0.05. Pearson’s correlation test was also performed to assess the linear correlation among the variables at *p* < 0.05.

## 3 Results and discussion

### 3.1 Model fitting

Based on the results of our study, it was observed that the studied variable responses varied depending on the three factors under investigation, namely maltodextrin (*X*1; %), gum Arabic (*X*2; %) percentage, and the concentration of the extract (*X*3; mg) (Table 1). The comparison of the TPC content of the 13 encapsulated extracts made evident a considerable degree of variability, as TPC values varied between 22 and 52 mg GAE/g DR in experiments 1 and 7, respectively. This signifies that the selection of factors employed for the encapsulation process plays a pivotal role in determining the phenolic content of the extracts. Furthermore, the antiradical capacity of the encapsulated extracts appeared to be highly sensitive to the ratio and composition of the coating materials utilized. For instance, the system incorporating the lowest concentration of extract in conjunction with maltodextrin and gum Arabic demonstrated remarkably high DPPH scavenging efficiency, reaching an impressive 74.5% of inhibition. Conversely, the system that contained the lowest extract concentration without the presence of gum Arabic had a limited scavenging ability (33%). A similar tendency was also observed for the particle size (Table 1), as the particle size of formulation 2 was 11 nm, which was the smallest of all the 13 experiments, whereas the size of particles of formulation 3 was 1,227 nm. In addition, the Pdi values of phenolic microcapsules were slightly larger in emulsion No 5. The Pdi values of emulsions 11, 12, and 13 were 0.503, 0.464, and 0.462, respectively, thus indicating that the particle structure was relatively polydisperse.

**Table 1:** Three-component axial screen matrix, with *X*1: maltodextrin percentage, *X*2: gum Arabic percentage, and *X*3: extract concentration ranging from 0.2 to 0.5, and the values of the experimental responses for TPC contents (*Y*1; expressed in mg GAE/g DR), antiradical activity (*Y*2; expressed in IP), *Y*3: particle size (*d*_3.2_ expressed in nm), and *Y*4: polydispersity index (Pdi)

Exp *N*′	*X*1	*X*2	*X*3	*Y*1	*Y*2	*Y*3	*Y*4
1	1.0000	0.0000	0.0000	22	33.46	359.0	0.533
2	0.0000	1.0000	0.0000	37.24	58.63	11.0	0.492
3	0.0000	0.0000	1.0000	24	51.39	1227.0	0.383
4	0.6667	0.3333	0.0000	51.11	74.54	123.44	0.161
5	0.3333	0.6667	0.0000	28.22	46.04	377.58	0.801
6	0.6667	0.0000	0.3333	34.8	46.62	911.23	0.535
7	0.3333	0.3333	0.3333	52.38	67.59	88.20	0.322
8	0.0000	0.6667	0.3333	35.57	60.51	881.54	0.461
9	0.3333	0.0000	0.6667	30	50.81	645.80	0.585
10	0.0000	0.3333	0.6667	25	40.4	640.7	0.619
11	0.6667	0.1667	0.1667	38.05	58.91	370.22	0.503
12	0.1667	0.6667	0.1667	32.84	54.14	1015.25	0.464
13	0.1667	0.1667	0.6667	39.82	61.81	501.69	0.462

### 3.2 Statistical analysis

NemrodW software package (Mathieu, Nony and Phan-Tan-Luu, version 2000, Marseille-France) was employed to calculate the model coefficients, to assess the variance analysis of fixed responses, and to develop mathematical and graphical analyses of the obtained results. The data obtained from the RSM were fitted to the second order polynomial equation, while the coefficients of regression of the independent variables and the corresponding *p*-values are presented in Table 2. The data of Table 2 underscore the significant impact of the studied factors (maltodextrin and Gum Arabic percentage and concentration of the extract) on the content of TPCs, antiradical capacity, size of the particles, and Pdi values of the encapsulated extracts. Moreover, the results highlighted the substantial variability in TPC content and the sensitivity of antiradical activity to the composition and ratio of coating materials utilized. Similar findings were observed for both particle size and Pdi parameters. The statistical significance of the regression model was assessed with the *F*-test using NemrodW software, and the results are also presented in Table 2. The obtained results of the validated mathematical model suggested that only independent variables recorded significant effects, as the regression coefficients of Fisher’s *F*-test were higher than the tabulated values, and the *p*-values were lower than 0.001. Table 2 also show that b3, b13, and b23 coefficients were significant regarding the droplet diameter. On the other hand, factors b1 and b2 were moderately significant, suggesting that the independent variables and their interactions significantly affected the targeted responses. Nevertheless, the impact of the interaction between the model variables was not significant as the regression coefficients of Fisher’s *F*-test were lower than the tabulated ones, and the *p*-value was larger than 0.001. Therefore, the regression equation suggested by NemrodW software should include the tested independent variables. According to Table 2 and for the variable response Pdi, all independent variables had a significant effect (*p* < 0.01). The analysis suggested that factors b3, b13, and b23 were significant. On the other hand, factors b1, b2, and b12 were not significant, as the regression coefficients of Fisher’s *F*-test were lower than the tabulated value, and the *p* value was higher 0.001.

**Table 2:** ANOVA results and mixture compound effects. Coefficient significance refers to emulsion variables and the studied responses (total phenolic content (TPC), DPPH test, size of particles, and polydispersity index (Pdi)) of the emulsions formulated with the peel squash refined extract

	TPC					DPPH test				
	Coefficient	*F*-inflation	Ecart-type	t.exp	Signif (%)	Coefficient	*F*-inflation	Ecart-type	t.exp	Signif (%)
b1	41.20	29.3	11.81	3.49	*	29.85	29.3	8.14	3.66	**
b2	30.84	29.3	11.81	2.61	*	49.40	29.3	8.14	6.06	***
b3	187.0	246.7	38.27	4.89	**	157.29	246.77	26.39	5.96	***
b12	51.01	2.11	18.19	2.8	*	11.67	2.11	12.54	0.93	38.69%
b13	−297.87	199.53	93.68	−3.18	*	−134.56	199.53	64.59	−2.08	7.4%
b23	−227.75	199.53	93.68	−2.43	*	−117.77	199.53	64.59	−1.82	10.9%
	**Droplet diameter**					**Pdi**				
b1	−0.485	29.3	0.098	−4.69	**	−0.299	29.30	0.187	−1.60	15.2
b2	−0.296	29.3	0.098	−3.03	*	−0.214	29.30	0.187	−1.14	29.1
b3	−3.579	246.77	0.316	−11.32	***	−3.272	246.77	0.606	−5.40	**
b12	0.35	2.11	0.150	2.33	5.1%	−0.009	2.11	0.288	−0.03	97.3
b13	10.01	199.53	0.774	12.94	***	9.102	199.53	1.482	6.14	***
b23	8.40	199.53	0.774	10.86	***	7.597	199.53	1.482	5.13	**

Significance: *p* < 0.01 (*), *p* < 0.01 (**) and *p* < 0.001 (***) mean very significant and strongly significant, respectively.

**Figure 1:**
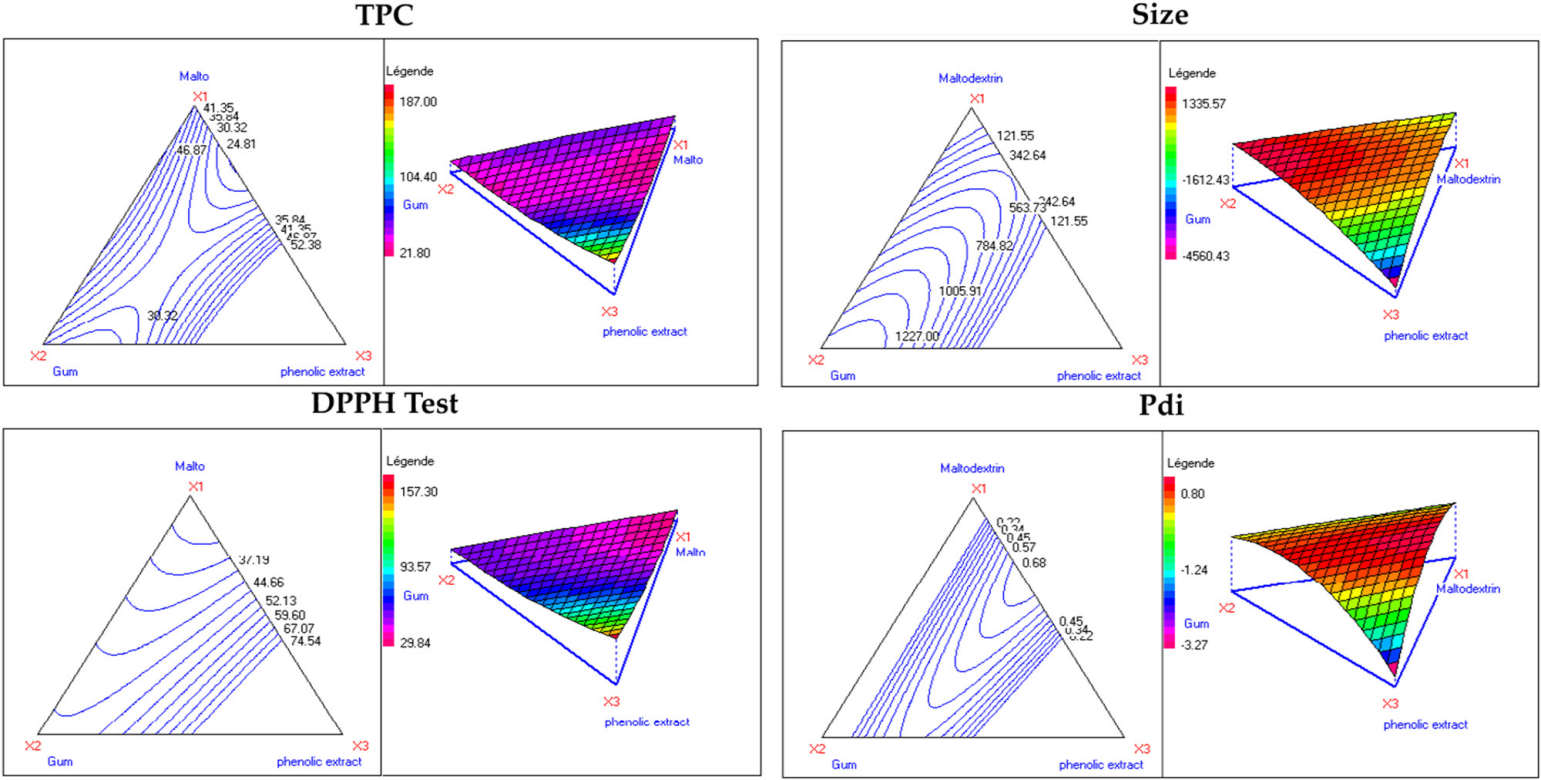
Isoresponse contours and mixture design space for emulsions formulated with the peel squash refined extract.

The coefficient of multiple determination (*R* ^2^) of 0.934 was calculated for the studied response (Pdi), showing a high correlation between the independent variables and the response. The suggested model was also statistically validated, further supporting its reliability. Besides, the graphical presentation of the *Y* response residuals (Figure 1) depicted the distribution of the points from the experimental and calculated values of the residuals. Figure 1 graphically presents the dimensional response surfaces of the significant interaction among the three independent variables for the particle size, size diameter, TPC, and antioxidant activity of the formulated emulsions. By examining the curvatures of the response surfaces and identifying the locations of maximal or minimal points for each response, we gain a much deeper understanding of the complex relationships within the emulsion formulation. This allows for a more targeted and effective approach to optimizing the emulsion properties by strategically selecting the proportions of different components.

#### 3.2.1 Mixture design plan

The implementation of maltodextrin and gum Arabic is one of the main factors of the encapsulation process. It is effective in maintaining the bioactive compound content and activity of the final product. The mixture compound effects on encapsulating polyphenols (Table 3) indicated that the coefficients of individual factors were significant parameters since their *p*-values were lower than 0.05, especially the refined extract concentration. In contrast, the individual factors exhibited highly significant antioxidant effects for the antiradical activity, while their interactions were not significant. Consequently, the predictive mathematical models, representing the response regarding the three independent variables, are represented by the following equations:

(3) \[\begin{array}{c}\text{For}\hspace{.5em}\text{TPC}:\hspace{.5em}{Y}_{\text{TPC}}=41.2\times X1+30.84\times X2\\ \hspace{7em}+187.0\times X3+51.01\times (X1\times X2)\\ \hspace{7em}-297.87\times (X1\times X3)\hspace{7em}-227.75\times (X2\times X3).\end{array}]\]

(4) \[\text{For}\hspace{.5em}\text{antioxidant}\hspace{.5em}\text{activity}:\hspace{.5em}{Y}_{\text{DPPH}}=29.85\times X1+49.40\times X2+157.29\times X3.]\]

(5) \[\begin{array}{c}\text{For}\hspace{.5em}\text{size}:\hspace{.5em}{Y}_{\text{size}}=-0.458\times X1-0.296\times X2\\ \hspace{7em}-3.579\times X3+10.015\times (X1\times X3)\\ \hspace{7em}+8.404\times (X2\times X3).\end{array}]\]

(6) \[\text{For}\hspace{.5em}\text{Pdi}:\hspace{.5em}{Y}_{\text{Pdi}}=-3.272\times X3+9.102\times (X1\times X3)+7.597\times (X2\times X3).]\]

**Table 3:** Experimental validation of the obtained formula

	Predicted value	Experimental value
TPC	47.46	46.01
DPPH test	66.54	64.85
Size	620.31	571.22
Pdi	0.52	0.49

These equations were transposed into isoprenic curves, as exhibited in Figure 1.

Altogether, the used software indicated that the targeted limit can be achieved with 91% desirability using a mixture consisting of

(7) \[23.8 \% \hspace{.5em}\text{Maltodextrin}+27.7 \% \hspace{.5em}\text{gum Arabic}+48.5 \% \hspace{.5em}\text{refined}\hspace{.5em}\text{extract}\text{.}]\]

The validation of this formula is detailed in Table 4.

**Table 4:** Basic physicochemical properties of the optimized formulation

Parameter (unit)	Result
DPPH test (inhibition %)	44.2 ± 2.04
ABTS test (inhibition %)	53.89 ± 0.07
Viscosity (mPa/s)	17 ± 0.01
pH	4.2 ± 0.23
Solubility (%)	93
Turbidity (NTU)	0.412
**Color**	
L*	102.4
a*	3.5
b*	7.8

Responses were measured in triplicate.

Antioxidant activity is usually associated with phenolic compounds [20], while significant correlations have been suggested between these two variables for the evaluated formulations [5,21] rendering the content of TPCs a useful indicator of their antioxidant capacity. Therefore, the proposed optimization of the extraction process should focus on increasing the recovering efficiency of phenolic compound content that eventually could improve the bioactive properties of the obtained matrix and its beneficial effects in food systems. Similar to our study, Stajčić et al. [22] suggested that the choice of appropriate encapsulation carriers and encapsulation mixture is pivotal to increasing the encapsulation efficiency of pumpkin waste, as well as to improve the bioactive potential of encapsulates. Moreover, Nooshi Manjili et al. [23] reported that multi-component hybrid encapsulation systems are more effective in protecting the bioactive compounds of pumpkin seed hydrolysates and in improving their release behavior and antioxidant capacity, while Rubio et al. [24] suggested the use of brewer’s spent yeast as an encapsulation carrier for carotenoids obtained from pumpkin peels. However, the encapsulation conditions should also be optimized through proper models, since they may also affect the efficiency of the process and the bioactive properties of the obtained encapsulates [25,26].

### 3.3 Physicochemical parameters and bioactive properties of the optimized emulsion

Once the obtained formulation was statistically validated, the optimized emulsion was characterized for various parameters either physicochemical (pH, viscosity, solubility turbidity, and color) or biological (antioxidant and antimicrobial activities).

#### 3.3.1 Color analysis

Color measurements included the parameters *L* (lightness) at 102.4, *a* (redness) at 3.5, and *b* (yellowness) at 7.8.

#### 3.3.2 Solubility

The solubility of the obtained microcapsules was 93%. Therefore, the dried formulation could be easily and instantly reconstituted when necessary.

#### 3.3.3 Viscosity

The viscosity of the optimized formula was 17 ± 0.01 mPa/s.

#### 3.3.4 pH

The pH of the extract was 4.2, showing a slight fluctuation of ±0.23.

#### 3.3.5 Turbidity

Turbidity was 0.412 NTU (Nephelometric Turbidity Units).

The data in Table 3 show a small droplet diameter (inferior to 500 nm) for the optimized emulsion, indicating that it possesses good stability. Moreover, the optimal droplet size distributions confirmed the stability of the emulsion since the experimental Pdi measurement was lower than 0.5. The measurement of the viscosity of the optimized formula showed a low value (17 mPa/s), confirming its stability since stable emulsions are characterized by low viscosity [27]. Indeed, the droplet size affected the viscosity of the emulsion, as it has already been suggested in oil-in-water emulsions [28]. The properties of the dispersed phase have a significant effect on the rheological properties of emulsions. The stability of the emulsion was also validated by the low viscosity, which matches with its small average droplet size [28]. In fact, the viscosity of the emulsion depends on the emulsion components (maltodextrin, gum Arabic percentage, and refined extract) and their concentrations.

Concerning pH measurement, our results suggested that the optimal emulsion had acidic nature with a pH value of 5.1. Moreover, our results were different from other works, which studied the formulation of several emulsions of phenolic rich extracts and recorded different pH values. These contradictory results could be associated with inorganic wetting that can reduce the tension of the surface of a liquid and consequently allow it to foam or penetrate solids in different formulations [29].

The turbidity values of the optimal formula indicated a turbidity of about 0.412 NTU. The turbidity varied depending on various parameters, including the mean particle size, particle concentration and Pdi values [30]. Besides, it was demonstrated that an emulsion with low turbidity was suitable to be integrated into optically transparent beverages or food products without affecting their optical properties [31].

Table 4 presents the values of L*, a*, and b* color parameters of the optimal formula. Both a* and b* values were low (3.5 and 7.8), a finding which indicates that the main color of the emulsion has a tendency to be yellowish. Emulsions generally are slightly turbid systems, because large particles scatter the light in a more intense manner than smaller ones. Therefore, as the droplet diameter of the refined extract enlarges, the scattering of light is higher, and emulsion is likely to be more opaque with increased lightness and whiteness [28,32].

## 4 Biological characterization

### 4.1 Antioxidant capacity

The optimized emulsion revealed its important antioxidant potential (Table 4). The ABTS values of 53.9% demonstrated significant radical scavenging ability. With a total antioxidant capacity of 4.2 mg GAE/g DR, the extract exhibited noteworthy capacity to counteract oxidants, which can have positive health implications. The content of TPCs of 37.2 mg GAE/g DR emphasized the abundance of antioxidative compounds in the emulsion. Additionally, the encapsulated extract’s antiradical activity of 44.2% underscored its efficacy in mitigating radical-induced damage. Despite minor fluctuations in some measurements, the overall findings strongly suggested that the encapsulated extract possesses robust antioxidant attributes.

### 4.2 Antibacterial capacity

**Figure 2:**
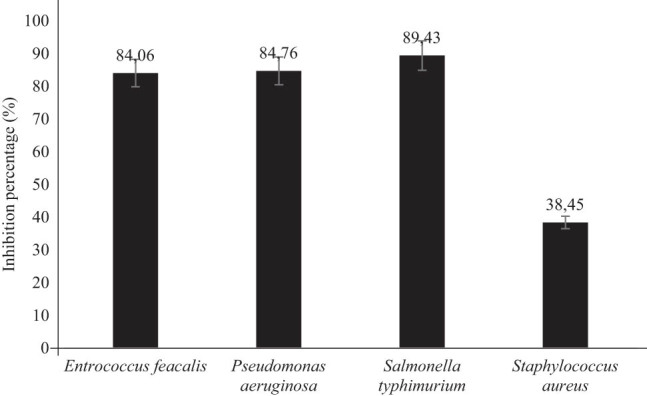
Growth IP of the optimized emulsion against various bacterial strains.

Figure 2 provides the growth IP of the optimized emulsion against various bacterial strains. *Enterococcus faecalis* exhibited an IP value of 84%, demonstrating its sensitivity to the tested agent. *Pseudomonas aeruginosa* also showed notable sensitivity with an IP value of 85%. *Salmonella typhimurium* recorded the highest sensitivity at 89%, whereas *Staphylococcus aureus* displayed a lower sensitivity with an IP of 38%. In our work, it was observed that the optimized emulsion exhibited fast inactivation of microorganisms. This behavior was demonstrated for *Salmonella typhimurium*, *Pseudomonas aeruginosa*, and *Enterococcus faecalis*, whereas the emulsion did not exhibit a significant inactivation of *Staphylococcus aureus*. The varied effectiveness of the optimized emulsion against the tested bacteria should be associated with the presence of specific compounds with antibacterial properties which may differ among the various genotypes of pumpkin [33,34]. Moreover, the composition of the encapsulation mixture may contribute to the overall bioactive properties of encapsulates, as already reported in the literature for various carriers [22,23,24], while the protocols used for the extraction of bioactive molecules from natural matrices such as pumpkin waste may also have a significant impact on the composition of the obtained encapsulates [22,23,24,35,36]. Finally, the physicochemical properties of encapsulates, such as droplet size and Pdi, have an effect on their stability and bioactive properties; according to Falleh et al. [31], droplets of small size and low viscosity may improve the stability and bioactive properties of encapsulates, while Aguilar-Toala et al. [37] suggested that low values of polydispersity index are associated with low aggregation of the obtained particles.

In general, the activity of phenolic compounds against microorganisms involves the interaction of these molecules with the proteins of the cytoplasmatic membrane that can precipitate and facilitate the leakage of ions and other cell contents resulting in the cell breakdown of the microbes [38]. In line with our results, many works showed a clear interaction between phenolic compounds and surfactant or biopolymer molecules through the microbial pores [39,40]. Phenolic compounds have significant antimicrobial impact through the degradation of membrane phospholipids and the consequent degradation of the cytoplasmic membrane. They are also capable of breaking down the outer membrane of Gram-negative bacteria, thus removing the lipopolysaccharides and increasing the permeability of the cytoplasmic membrane. These compounds are also able to regulate the growth and the production of toxins from pathogens [41,42].

## 5 Conclusions

The Box Behnken design was effectively used for the optimization of the emulsification process of the extracted phenolic compounds from the peels of squash fruit. The obtained optimized encapsulation conditions, e.g. maltodextrin percentage of 23.8%, gum Arabic percentage of 27.7% and phenolic extract percentage of 48.5%, resulted in an emulsion of high quality with high TPC content and high antiradical and antimicrobial capacities. Our results also suggested that the experimental values of TPC, DPPH test, particle size, and Pdi were in accordance with the ones predicted from the model, thus confirming the potential for developing microcapsules of high quality and their integration in novel food and pharmaceutical products. In conclusion, further studies should be performed using pumpkin peels rich in phenolic compounds to encourage consumer acceptability, aiming to valorize this particular biowaste and to increase the added value of this valuable crop, in accordance with the concept of circular economy. Moreover, future research should focus on the evaluation of the properties of the optimized emulsions under food processing techniques (e.g., pasteurization) and storage conditions to validate their potential integration into real food systems.
